# Case-control study on associations of hemorrhagic bowel syndrome in swine with feed characteristics and intestinal pathogens

**DOI:** 10.1186/s40813-024-00397-3

**Published:** 2024-10-19

**Authors:** Fabienne Holenweger, Peter Spring, Negar Khayatzadeh, Andreas Hofer, Gertraud Schüpbach-Regula, Alexander Grahofer

**Affiliations:** 1 Clinic for Swine, Department for Clinical Veterinary Medicine, Vetsuisse Faculty, University of Bern, Bern, Switzerland; 2 Berne University of Applied Sciences, HAFL - Agricultural Sciences, Zollikofen, Switzerland; 3 SUISAG, Allmend 10, Sempach, 6204 Switzerland; 4 Department of Clinical Research and Veterinary Public Health, Vetsuisse Faculty, Veterinary Public Health Institute, University of Bern, Bern, Switzerland

**Keywords:** Sudden death, HBS, Haemorrhagic bowel syndrome, Swine, Feeding system, Feed hygiene, B. pilosicoli

## Abstract

Haemorrhagic bowel syndrome (HBS) is one of the most common causes of death in fattening pigs worldwide. The objective of this descriptive study was to systematically assess predictors or causal components for the appearance of HBS using case farms (mortality rate caused by HBS ≥ 1.5%) in comparison with control farms (mortality rate caused by HBS ≤ 0.25%), focusing on feed ingredients, feed quality and size, and gastrointestinal pathogens. The inclusion of sugar beet as a feed component in liquid feeding systems was found to be associated (*p* = 0.03) with farms identified as HBS cases. Another predictive or causal factor found for liquid feeding systems, but only for those using meal, was particle size. A higher percentage of small particles (< 2 mm) in the meal was associated with a higher risk of being an HBS case farm (*p* = 0.02), while no relevant association was detected for the use of pellets. Sugar beet in the diet was also associated with the incidence of HBS.

The microbial quality of the feed in dry feeding systems, specifically the number of total aerobes at the first and last outlet tubes, was associated with a higher incidence of HBS (*p* = 0.03). Faecal sample analysis showed a difference (*p* < 0.05) in the prevalence of *B. pilosicoli *in the herd category (case vs. control herd). In this descriptive study, five predictive or causal factors were identified for an HBS farm with a mortality rate due to HBS ≥ 1.5%. These included the number of aerobes in dry matter samples from the first and last feeders, the particle diameter of the meal used in liquid feeding systems and sugar beet as a component of liquid feeding rations, and the presence of *B. pilosicoli* as an infectious agent at animal level. Relevant associations reinforce the findings of the previously published Swiss study that HBS is a multifactorial syndrome involving different aspects of pig production and cannot be attributed to a single cause. Further studies are needed to develop evidenced based causal models for HBS in swine.

## Introduction

One of the main causes of sudden death in pigs, most often affecting pigs from 2 to 6 months of age, is the haemorrhagic bowel syndrome (HBS) [1, 2]. The syndrome is characterised by the absence of any premonitory clinical signs prior to acute death. When pigs are necropsied after death, a well-filled stomach and distended and haemorrhagic intestines may be seen, oftenassociated with a torsion of the mesenteric root in a counterclockwise direction of at least 180 degrees from a ventro-caudal aspect [3]. Besides the significant economic impact on pig farms due to the loss of pigs, the animal welfare impact of HBS cannot be neglected [4]. In addition, the unpredictable mortality rates, which vary considerably between groups, place a further burden on farmers. Mortality rates per group are often in the range of 0–7%, without the affected pigs showing any clinical signs prior to death [5]. The causation of HBS as a syndrome in pigs is not yet fully understood, although the the syndrome has been described in pig herds worldwide. The most commonly reported factors associated with HBS are liquid feeding systems with whey as part of the diet, but the syndrome can also be seen in herds with dry or wet-dry feeders [5, 6]. Liquid feeding of pigs is practised worldwide because of its potential to use by-products from the food industry. In Switzerland, a large amount of whey is produced as a by-product of cheese production, which can be reused in pig production [7]. Pelleted feed is mentioned as a risk factor by several authors [8, 9]. A Swiss case-control study identified several housing and management related risk factors for HBS in growing-finishing pig herds [10]. The sire breed of the fattening pigs, the number of origins of pigs per batch, the frequency of cleaning of the feed distribution system and the feeding space per finishing pig were significantly associated with the incidence of HBS. This indicates that HBS is a multifactorial issue influenced by various aspects of pig husbandry and production. To date, farms with dry or spot-mix feeding systems have not been included in scientific studies.

The influence of feed composition, feeding system, and hygiene of the feed and feeding system on sudden death in pigs with distended abdomen has been described previously [11]. In a similar direction, a Swiss case report [3] investigated a grower-finisher farm with a sudden increase in mortality due to HBS. Both authors worked with farms that had implemented liquid feeding systems in their production, differing only in feed composition and distribution method and frequency. In the 1990 study, various case farms were compared to identify common factors [11]. The rations fed on the farms were rich in different saccharides and water, providing good conditions for the development of yeasts and bacteria. Furthermore, the liquid rations were generally low in fiber, but rich in minerals. A high level of contamination of the feed with microorganisms, including yeasts, was found after passing through the feeding system. In other farms, contamination had already occurred at the level of the storage tanks, leading to gas formation. In the Swiss study [3], the main findings were a pH value of 5.0 in the liquid feed in the mixing tank and high levels of *Enterobacteriaceae* in the liquid feed samples. The *Enterobacteriaceae* levels measured in colonies-forming units per gram (CFU / g) were 140 to 150 times higher than the reference values proposed by Kamphues [12], whereas other parameters measured in the same samples (i.e. *Escherichia coli* and yeast) were within the reference values [12, 13]. Not only the feeding systems can be affected by the increased gas production, but also the intestinal system itself. Highly fermentable diets as well as overfeeding and abnormal fermentation can lead to gas formation in the small and large intestine [13]. This lead to increased pressure in the intestines and the abdomen in general, and an increased risk of torsion [13, 14]. Whey and other carbohydrate-rich fluids are examples of highly fermentable components of rations commonly used in the pig industry. As part of the dry matter, soya is often used in pig production due to its higher protein content and better amino acid balance compared to other plant-based protein sources [15].

The hygiene of the feeding system has been reported to have a significant impact on the incidence of HBS, and other gastrointestinal diseases, especially in liquid feeding systems. Two studies from France investigated farms with sudden deaths in healthy finisher pigs [16]. After a thorough investigation of the farms, a new cleaning protocol for the feeding system was established, which did not lead to an overall reduction in the incidence of HBS. However, in some farms an increased incidence of HBS was observed immediately after the cleaning of the feeding system [17]. The results of both studies provided the basis for other authors to further investigate the influence of cleaning and disinfection on feed hygiene and the development of a new flora in cleaned systems [18]. Conclusions of the study were that the term contamination of a feeding system should be interpreted with caution, as the flora found in samples taken before and after cleaning (i.e. lactic acid bacteria and yeast) is consistent with the physiological flora of the feed. Similar findings were described in a study from Denmark [19] after cleaning of the feeding system, but with regard to the occurrence of diarrhoea instead of HBS.

A possible infectious agent as a cause of HBS or as an associated factor could not be excluded [5, 20]. For example, a toxicity test at 8–10 weeks of age by intravenous injection of filtered intestinal content from pigs that died of HBS, as well as by oral administration in different pigs, did not lead to HBS in the exposed animals [4]. The most common bacteria implicated in HBS are are *Lawsonia (L.) intracellularis*, *Brachyspira (B.)* spp., *Escherichia coli* and *Clostridia spp* [5, 20]. Sudden deaths in pigs caused by infection with *L. intracellularis* or *B. hyodysenteriae* could be misdiagnosed post mortem as HBS pigs due to the presence of bloody intestinal contents and discolouration of the intestinal walls [21]. *L. intracellularis* has been suspected as the causative agent of HBS by South African swine farmers and veterinarians [22]. An survey of South African pig farms with a known HBS problem showed that typical lesions of porcine hemorrhagic enteritis (PHE) caused by *L. intracellularis* could not be deteced at necropsy [22]. Furthermore, the clinical presentation of HBS and PHE is different. The first clinical signs observed in PHE-affected pigs are bloody diarrhoea and not sudden death of the pig, as seen in HBS. Other authors have hypothesize that *L. intracellularis* in combination with toxin producing bacteria, such as *E. coli* and *C. perfringens*, may lead to peracute death consistent with HBS [5]. The presence of swine dysentery caused by *Brachyspira* has also been suggested as a possible pathogen associated with HBS. In a study from the Slovak Republic, this could not be confirmed, because *B. hyodysenteriae* was not detected in the intestines or in the serological screening for antibodies [5].

Therefore, the main objective of this descriptive study was to analyse associations between HBS in Swiss fattening farms with feed characteristic in order to generate hypotheses regarding feed-related predictors or causal components. As a secondary objective, the presence of *L. intracellularis*, *B. hyodysenteriae* and *B. pilosicoli* in the faecal samples was analysed to investigate an association between infectious agents and the incidence of HBS.

## Results

In total, the response rate of the farmers was 72%. Farmers of 18 herds (15 control and 3 case herds) were not reachable after calling twice and leaving a message. 7 control herds and 13 case herds didn’t want to participate in the study. This was due to lack of interest, COVID- infection or suicide. In addition, 19 farms (3 control and 16 cases) no longer met the requirements of the study.

Overall, 315 feed samples (liquid feed = 232, dry feed = 83) and 187 water samples of 97 herds (48 case herds and 49 control herds) were collected for the microbial analysis. Furthermore, on 68 farms (34 case and control herds each) a dry feed sample was taken for the particle size analysis, respectively, and each of the 97 farms visited provided a detailed list of feed components. Due to COVID-19 related restrictions and/or cancellations shortly before the farm investigation, three herds had to be excluded. Herds that housed exclusively grower and finisher pigs made up the majority (*n* = 85), only a few (*n* = 12) were closed herds with other age groups in the control and case population, respectively.

### Feed components

For the feed components, the characteristic was binary (i.e.present yes/no) and was therefore not changed for the statistical analysis (Tables 1 and 2). The omponents were analysed independently of the type of feeding system, where sugar beet, soybean meal, and lactose showed an association (*p* < 0.1) with being from a HBS farm. The analysis was also carried out after dividing the farms into two groups according to the feed system used on the farm. For liquid feeding systems, sugar beet, soybean meal, and lactose showed an association with belonging to HBS farms. In addition, potato protein and cheese scrapes (cheese industry residues, non-liquid) as part of thediet were associated to case farms with liquid feeding systems. In contrast to this, only potato protein was associated with HBS cases using a dry feeding system. The feed components in the liquid diet were analysed by univariable models and five of the 32 variables were associated to being an HBS case farm (i.e. sugar beet, soybean meal, lactose, potato protein, and cheese scrapes). All these components were not correlated with each other, and therefore were included in a multivariable analysis. Sugar beet was the only variable associated with HBS (*p* = 0.03) in the multivariable model. Potato protein was associated with HBS case farm with a p-value of 0.08.

**Table 1:** Descriptive statistic and univariable analysis (logistic regression) of the feed components in the feeding regime working with liquid feed system grouped by case and control herds

Liquid feeding system	Case herds	Control herds	Odds ratio	Lower 95% confidence limit	Upper 95% confidence limit	*p*-Value
Sugar beet (%)	90.9	68.7	4.5	1.1	18.5	0.02
Soybean meal (%)	100.0	87.5	* not applicable	* not applicable	* not applicable	0.02
Permeat / Lactose (%)	0.0	9.1	* not applicable	* not applicable	* not applicable	0.04
Potato protein (%)	18.7	3.2	6.9	0.8	61.3	0.04
Cheese scrapes (%)	8.8	0.0	0.3	0.3	0.3	0.05

**Table 2:** Descriptive statistic and univariable analysis (logistic regression) of the feed components in the feeding regime working with dry feed system grouped by case and control herds

Dry feeding system	Case herds	Control herds	Odds ratio	Lower 95% confidence limit	Upper 95% confidence limit	*p*-Value
Sugar beet (%)	78.6	66.7	1.8	0.3	9.7	0.47
Soybean meal (%)	100.0	93.3	* not applicable	* not applicable	* not applicable	0.25
Permeat / Lactose (%)	0.0	0.0	* not applicable	* not applicable	* not applicable	**-**
Potato protein (%)	7.7	37.5	0.1	0.0	1.4	0.05
Cheese scrapes (%)	0.0	6.2	* not applicable	* not applicable	* not applicable	0.05

### Particle size feed

For the description and statistical analysis, four groups were created depending on whether they came from farms with pellet versus meal feed and whether a liquid vs. a dry feeding system was implemented on the farm. Furthermore, for visualisation, the results were dividedinto eight groups by case/control, pellet/meal, and liquid/dry feeding systems (Table 3). Farms with liquid feeding systems containing meal feed showed an association (*p* < 0.1) for small particles from an HBS case farm (Table 3). Liquid feeding systems with pellets as well as both categories of dry feeding systems did not show an association with HBS, neither for small particles nor for coarse particles.

**Table 3:** Descriptive statistic and univariable analysis (logistic regression) of the particle size examination of the feed samples of 68 farms (2 samples per farm) grouped by case/control, shape and feeding method

Pellets / Liquid feed	Case herds (Feed samples *n* = 4) Median (min-max)	Control herds (Feed samples *n* = 10) Median (min-max)	Odds ratio	Lower 95% confidence limit	Upper 95% confidence limit	*p*-Value
< 4 mm	90.1% (82.4–97.0)	97.2% (89.5–100.0)	* not applicable	* not applicable	* not applicable	0.07
< 2 mm	46.1% (11.9–80.4)	72.6% (43.7–94.4)	54.6	0.4	8264.0	0.1
< 1 mm	21.6% (3.8–39.9)	28.5% (13.7–46.4)	* not applicable	* not applicable	* not applicable	0.4
< 0.5 mm	10.3% (2.0-19.6)	11.8% (6.7–21.2)	* not applicable	* not applicable	* not applicable	0.7
< 0.18 mm	0.4% (0.2–0.6)	1.2% (0.4–3.9)	* not applicable	* not applicable	* not applicable	0.4
< 4 mm	66.0% (2.5–99.4)	73.8% (4.5–99.5)	1.5	0.1	18.7	0.7
< 2 mm	56.5% (1.8–85.9)	42.4% (4.0-98.8)	0.4	0.0	6.3	0.5
< 1 mm	28.6% (1.4–47.7)	14.3% (1.9–27.2)	0.0	0.0	5.9	0.1
< 0.5 mm	13.3% (1.1–20.4)	6.4% (1.0-11.7)	0.0	0.0	21.8	0.1
< 0.18 mm	0.9% (0.4–1.7)	0.6% (0.2-1.0)	* not applicable	* not applicable	* not applicable	0.2
< 4 mm	96.7% (80.2–100.0)	97.9% (93.2–100.0)	* not applicable	* not applicable	* not applicable	0.1
< 2 mm	80.8% (26.6–98.9)	88.2% (64.8–99.1)	33.0	1.2	948.0	0.04
< 1 mm	38.9% (8.6–81.9)	48.7% (16.2–85.2)	14.7	1.5	143.2	0.02
< 0.5 mm	17.5% (3.0-43.5)	20.3% (1.7–44.0)	15.8	0.2	1020.0	0.2
< 0.18 mm	1.6% (0.2–5.1)	2.0% (0.2–9.8)	* not applicable	* not applicable	* not applicable	0.3
< 4 mm	98.9% (97.7–99.6)	96.1% (84.9–99.6)	* not applicable	* not applicable	* not applicable	0.4
< 2 mm	83.4% (69.6–97.0)	75.3% (24.1–98.3)	0.1	0.0	22.7	0.4
< 1 mm	30.6% (15.5–41.9)	34.6% (10.9–65.2)	6.3	0.0	3758.3	0.6
< 0.5 mm	14.1% (6.2–25.2)	15.3% (4.5–35.4)	* not applicable	* not applicable	* not applicable	0.8
< 0.18 mm	1.3% (0.5–2.6)	2.7% (0.3–11.2)	* not applicable	* not applicable	* not applicable	0.3

In addition, the results of the particle size distribution analysis were divided into two groups by diameter, coarse particles < 2 mm in diameter and small particles < 2 mm in diameter. In the multivariate model of particle size analysis, the group of small particles < 2 mm indiameter showed a relevant association with the incidence of HBS (*p* = 0.02). This finding was only described for farms with a liquid feeding system that included meal. All other groups, i.e. the liquid feeding system with pellets, as well as the dry feeding systems with meal or pellets, showed no significant correlation of either of the two diameter groups with being a HBS case farm.

### Microbiological investigations feed and water

Microbiological methods were used to analyse the feed and water samples and determine the amount of bacteria. Feed samples were also analysed for yeasts and moulds. The results, expressed as colony-forming units per gram of feed (CFU/g), were logarithmically transformed for the statistical analysis and therefore retained as numeric variables (Tables 4 and 5). Overall, in the liquid feeding system, 87% of samples were above the reference for the first total aerobs drainpipe, 18% were above the reference for the first Enterobacteriaceae drainpipe, and 33% were above the reference for the first yeast drainpipe. For the last drainpipe, 88% are above the reference for total aerobes and 24% above the reference for *Enterobacteriaceae*. The *E. coli *measured in the whey exceeded in 9% of the samples the reference value.

**Table 4:** Descriptive statistic and univariable analysis (logistic regression) of the microbiological examination in the feeding regime working with liquid feed system grouped by case and control herds

Liquid feeding system	Case herds (*n* = 34) Median (min-max)	Control herds (*n* = 33) Median (min-max)	Odds ratio	Lower 95% confidence limit	Upper 95% confidence limit	*p*-Value
Total aerobs first drainpipe	16.6 (9.9–21.4)	16.6 (11.3–24.4)	1.0	0.8	1.2	0.92
Enterobacteriaceae first drainpipe	5.5 (2.3–11.9)	5.4 (2.3–11.7)	1.0	0.8	1.1	0.87
Yeasts first drainpipe	9.5 (2.3–16.6)	10.6 (4.6–15.5)	* not applicable	* not applicable	* not applicable	0.16
Total aerobs last drainpipe	16.6 (9.8–19.8)	16.7 (11.8–19.5)	1.0	0.8	1.2	0.96
Enterobacteriaceae last drainpipe	5.7 (2.3–11.9)	5.3 (2.3–11.9)	1.0	0.8	1.1	0.64
E.coli whey	2.3 (2.3–9.9)	2.3 (2.3–2.3)	* not applicable	* not applicable	* not applicable	0.04

**Table 5:** Descriptive statistic and univariable analysis (logistic regression) of the microbiological examination in the feeding regime working with dry feed system grouped by case and control herds

Dry feeding system	Case herds (*n* = 34) Median (min-max)	Control herds (*n* = 33) Median (min-max)	Odds ratio	Lower 95% confidence limit	Upper 95% confidence limit	*p*-Value
Total aerobs first drainpipe	9.8 (8.0-12.6)	11.4 (8.0-15.6)	1.7	1.0	2.8	0.01
Enterobacteriaceae first drainpipe	3.4 (2.3–8.1)	5.9 (2.3–11.9)	1.4	1.0	2.0	0.02
Yeasts first drainpipe	2.8 (2.3–4.6)	4.4 (2.3–14.7)	* not applicable	* not applicable	* not applicable	0.09
Total aerobs last drainpipe	9.8 (8.7–12.7)	11.3 (8.1–15.6)	1.8	1.0	3.2	0.02
Enterobacteriaceae last drainpipe	3.0 (2.3–7.3)	5.1 (2.3–11.9)	1.3	0.9	1.9	0.05

In the dry feeding system, 5% of the samples in the first drain pipe were above the reference for total aerobes, 0% for *Enterobacteriaceae* and 7% for yeasts in the first drain pipe, respectively. For the last drain pipe, 7% exceeded the reference for total aerobes and *Enterobacteriaceae* were within the reference. Grouped by feeding system, the results of the microbiological analysis of farms with liquid feeding systems were above the reference for total aerobes in 87% of the samples from the first drain pipe and 88% of the samples from the last drainpipe, for *Enterobacteriaceae* 18% of the samples from the first drain pipe and 24% of the samples from the last drain pipe exceeded the reference. Yeast contamination measured was significantly more common in liquid feeding systems, with 33% of the first drain samples and 27% of the last drain pipe exceeding the reference value used For the whey sampled, 9% of the farms had values above the reference value. For farms with dry feeding systems, the results from the microbiological analysis were above the reference for total aerobes in 5% of the first drain pipe samples and 7% of the last drain pipe samples, for *Enterobacteriaceae* all the samples of the first and the last drainpipe, respectively, were within the reference value.

For the dry feeding system, two different models were realised, one for the first drain pipe and one for the last drain pipe. In the model of the first drain pipe, the number of total aerobes was shown to be associated with the incidence of HBS with a p-value of 0.03, for *Enterobacteriaceae* at the same sampling spot, an influence on the total aerobes value could be seen, but no association with HBS farms. The same observation could be made with the values of the last drainpipe, where the p-value of total aerobes was 0.03 and *Enterobacteriaceae* had an influence on total aerobes without being significantly associated with HBS. Yeast did not show a significant association to HBS farms or a tendency to have an influence on the disease complex. Furthermore, the amount of *E. coli* in the whey samples was associated with the incidence of HBS with a p-value of 0.04 but as a protective factor rather than of a risk factor.

### Analysis of fecal samples

Fecal samples collected on the farms were analysed by biochemical methods and results were reported for each individual sample. For further analysis, the individual results were used as a binary variables (i.e., positive / negative) and as a continuous variables (i.e. logarithmic GE/g for *L. intracellularis* and ct value for *B. hyodysenteriae* and *B. pilosicoli*) at the animal level and as a binary variable (i.e., positive / negative) at the farm level. None of the samples were positive for *B. hyodysenteriae*, therefore only the results for *L. intracellularis* and *B. pilosicoli* were further described in Tables 6 and 7. Analysis of the faecal sample showed a difference (*p* < 0.05) of the prevalence of *B. pilosicoli* and the herd category. A higher prevalence of *B. pilosicoli* could be detected in case herds. *L. intracellularis* showed no significant correlation in the univariablee analysis, neither for the presence of bacteria nor for the measured equivalents of the genome on the animal level. No association was found at farm level for either of the two bacteria.

**Table 6:** Descriptive statistic and chi-square test of the biochemical analysis for *Lawsonia intracellularis* of the faecal samples grouped by case and control herds

*Lawsonia intracellularis*	Case herds	Control herds	*p*-value
n farms positive / total tested	3/13	4 /13	0.66
n animals positive /total tested	9 / 130	15/130	0.2
log genome equivalents /g faeces Median (Min/Max)	0.39 (0-6.4)	0.82 (0-9.1)	0.15

**Table 7:** Descriptive statistic and chi-square test of the biochemical analysis for *Brachyspira pilosicoli* of the faecal samples grouped by case and control herds

*Brachyspira pilosicoli*	Case herds	Control herds	*p*-value
n farms positive / total tested	5 /13	5 /13	1
n animals positive /total tested	20 / 130	10/130	0.05
ct-value Median (Min/Max)	5.55 (0-39.5)	2.72 (0-38.5)	0.05

## Discussion

The aim of the present case-control study was to describe feed-related predictors or causal components and potential infectious agents that contribute to the incidence of HBS in pigs on Swiss fattening farms. To achieve this objective, 97 Swiss fattening farms were investigated, 48 HBS case farms with a mortality rate of ≥ 1.5% to HBS within one year and 49 control farms with mortality rate of due to HBS ≤ 0.25% within one year. Potential selection bias was minimised by a high response rate of 72% and selecting farms solely on the mortality rate of HBS within one year. This approach ensured that our sample accurately reflects the source population’s variation in HBS incidence. By capturing a range of mortality rates, we consider our sample is representative of the broader target population. The time point of the investigation was not necessarily at the time when a case of HBS occurred on the farm, so for example, the quality of the feed may have been different at the time when the cases occurred.Similar to the fecal samples, the pigs were randomly selected for sampling. No data was collected regarding potential HBS cases within the same group or the subsequent mortality of the sampled pigs due to HBS. Overall, the univariate analysis revealed that six variables from the microbiological analysis were significantly associated with being from an HBS case farm. Additionally, five variables from the feed component analysis and one variable from the particle size analysis showed significant associations.Additionally, *B. pilosicoli* was identified in the fecal sample analysis showed a significant correlation with being on an HBS case farm when analyzed at the animal level, but not at the herd level. The variables from both the microbiological analysis and the feed components were incorporated into multivariate modelling and analysed separately for liquid and dry feeding systems.The influence of feeding systems and feed hygiene on the incidence of HBS in fattening pigs has been previously discussed. However, prior studies focused exclusively on liquid feeding systems, leaving a gap in understanding the situation in herds with dry feeding systems, including newer systems that mix dry components with water directly at the animal level (e.g., spot mix systems).

The speculation by other authors that HBS is caused by contamination of the liquid feeding system could not be confirmed in this study. None of the sampling sites or bacterial analyses from the liquid feeding systems showed a significant association between the case and control herd. Instead, feed hygiene in dry feeding systems was found to be crucial for the incidence of HBS. Significant associations were observed between the number of total aerobes at the first and last drain pipe/feeder and the presence of HBS in comparison to the control herds. Additionally, there was a tendency for increased risk of HBS with higher numbers of* Enterobacteriaceae* in the dry feed samples.

These findings suggest that feed hygiene in dry feeding systems is more critical than previously recognized. One possible explanation is the prolonged duration that feed remains in the feeder containers and distribution systems, resulting in a longer time from storage to consumption compared to liquid feeding systems. Moreover, dry feeding systems are more susceptible to contamination from dust and fecal particles from the pens. The warm and often humid air in the pens provides an ideal environment for bacterial growth in the feed. This issue can be exacerbated by not properly closing the feeder flap, which was commonly observed in the fattening pens studied.

Furthermore, the use of whey in the ration, without consideration of its microbial quality, did not show a significant correlation with HBS in Swiss fattening farms.

 [11]. When analyzed microbiologically for total aerobes, *E. coli*, *Enterobacteriaceae*, yeasts, and moulds, the whey from HBS case farms exhibited significantly higher concentrations of *E. coli* compared to that from HBS control farms. The potential protective role of *E. coli* in whey with regard to HBS is challenging to explain. One hypothesis is that higher *E. coli* concentrations might increase competition for substrates, thereby reducing the availability of nutrients for other potentially gas-forming bacteria. Additionally, the amount of *E. coli* may be reduced in the stomach due to the acidic environment, which generally mitigates its potential risk to pig health [23].

The particle size of the feed is known to have an impact on the health of the gut and the growth performance of fattening pigs and was studied by different authors [23–25]. The association between particle size and the occurence of HBS has not yet been investigated, but rather the appearance of stomach ulcers and the influence on the intestinal microflora. In a study a correlation was found between particle size and amount of *Enterobacteriaceae* in the caecum, distal small intestines, and mid colon. In addition a coarser diet (diameter < 5 mm) had a reduced *Enterobacteriaceae* count in specific intestinal sections compared to a fine diet (diameter < 2 mm) [23]. This observation suggests that a coarser diet, with a higher percentage of particles larger than 2 mm, might compensate for hygiene deficiencies in the feed. In the present study, liquid feeding systems using meal showed an increased risk of HBS when the percentage of particles smaller than 2 mm increased. This supports the hypothesis that larger feed particles may help reduce bacterial levels in the gastrointestinal tract.A similar effect could not be seen with liquid feeding systems using pelleted feed, where neither of the two particle diameter groups (that is, diameter 2 mm and diameter < 2 mm) showed a significant correlation with HBS. Dry feeding systems appear to be neither negatively nor positively impacted by the particle size of the feed in use with respect to the incidence of HBS. Further investigations into the association of *Enterobacteriaceae* in the feed and intestinal contents, as well as the impact of particle size in the feed, on the occurrence of HBS could provide valuable insights.

All components of the different feeds and mixtures used in the examined farms were examined and analysed. The univariate analysis resulted in five components that were significantly associated with HBS in farms where a liquid feeding system was used. In the multivariate analysis, sugar beet was the only feed ingredient with a significant association to the incidence of HBS. Potato protein was not significantly associated with the HBS herds in comparison with the control herds but showed a tendency to influence the incidence of HBS. Sugar beet is often used in rations due to its high availability on the market and its high crude fibre content, but it is also easily fermentable by the pigs’ microflora [12].

Due to the high content of easily fermentable components, increased gas production in the intestines may occur, potentially causing the intestines to become displaced and move more freely within the abdominal cavity [6, 13]. The potential influence of potato protein on the occurrence of HBS should be interpreted with caution due to the small number of farms (15%) using it in their rations. Further investigation is needed to better understand its impact.

The fecal sample analysis using qPCR revealed that Brachyspira pilosicoli had a significant impact on classifying the herd as HBS when assessed at the individual animal level. This result must be interpreted with caution due to the small sample size relative to the total number of farms and animals examined. Additionally, no follow-up information was collected on the sampled animals, leaving it unclear whether any of them subsequently died from HBS during the remainder of the fattening period. Sampling the intestinal contents of pigs that have died from HBS for biochemical analysis could be a potential approach to further investigate the effect of infectious agents on HBS. These results could then be compared with samples from fattening pigs at slaughter and/or pen-mates of the affected pigs. At farm level, no association was found between *Brachyspira pilosicoli* and *Lawsonia intracellularis* and HBS. In addition,* L. intracellularis* did not have a significant effect on HBS at the individual animal level.

## Conclusion

The present study is one of the first case-control studies with the aim of systematically identifying feed-related predictors or causal components for the incidence of HBS in fattening farms. Furthermore, the study examined the association between infectious agents and HBS at the herd and animal level. In this descriptive study, five predictive or causal factors including the number of aerobes in dry feed samples from the first and last feeder, the particle diameter of meal used in liquid feeding systems and sugar beet as component of liquid feeding rations, as well as the presence of *B. pilosicoli* as infectious agent at the animal level, were identified for an HBS farm with a mortality rate due to HBS ≥ 1.5%.

The significant associations found in this study support the findings of our previous study that identified HBS as a multifactorial syndrome affecting various aspects of fattening pig production. This syndrome cannot be attributed to a single cause and involves factors such as environment, management practices, feed hygiene, feed components, and gastrointestinal tract infections.

To reduce mortality rates on farms affected by HBS and improve economic efficiency and animal welfare, a comprehensive investigation of individual farms—including the risk factors identified—is essential. The findings of this study can help to develop tailored management practices covering feed, environment, and housing. Successful implementation will require strong collaboration among farmers, veterinarians, marketers, feed mills, and feed technicians to mitigate the impact of this multifactorial syndrome.

Even though we cannot establish any causal factors for HBS, our study is nevertheless important for generating hypotheses regarding feed related risk factors. This can be used as a basis for future experimental studies testing specific hypotheses.

## Materials and methods

The present work is part of the study described in: Holenweger, F., Schüpbach, G., Hofer, A. et al. Housing and management factors and breed predisposition for haemorrhagic bowel syndrome in swine. Porc Health Manag 9, 44 (2023). https://doi.org/10.1186/s40813-023-00340y [10].

### Ethical approval

The protocol of this study was approved by the Berne Cantonal Veterinary Office (license number BE48/2021; 33799).

### Study design and herd selection

This descriptive case-control study included 100 Swiss pig herds. Potential herds were selected with the help of the SUISAG Pig Health Service and its database, which includes records from 490.000 fattening units. Data included herd size, an electronic pig mortality log with with reason for mortality, and a treatment journal.

50 herds with a mortality rate of 1.5% or more due to HBS within one year, as well as 50 herds with a mortality rate of 0.25% or less due to HBS, were selected as case and control herds, respectively. Only herds with 600 or more finisher pigs slaughtered per year were included in this study. SUISAG contacted the farms that met the inclusion criteria and asked for permission to give contact information to the Co-Investigator of this study. By telephone, the Co-Investigator contacted 154 farmers and obtained more detailed information from the farmers about the study and the procedures, as well as a verification of the mortality rate. Each farm was visited once. At the time of the visit, each farmer signed an agreement for the use of the data and samples collected from their farm, as well as a declaration of freedomfrom highly contagious diseases. All data were anonymized before analysis. During the farm visit, a questionnaire was completed with information on feed and herd management, as well as general information on the herd during a personal interview. The questionnaire was validated by a visit to 3 farms; data from these investigation were not included in the analysis. Herds that did not record their losses or did not give a reason for the losses were excluded from the study. Only farms with no changes in housing, feed or breeding of the fattening pigs during the 6 months prior the investigation were included. The fattening pigs were divided into two groups, grower pigs (25–60 kg body weight) and finisher pigs (61–120 kg body weight) in order to account for the differences in the feeding regimes established in most Swiss fattening farms.

The sample size of 50 case and 50 control herds, respectively, ensured 80% power to detect odds ratios greater than 3.5 at the 5% significance level when the prevalence of the risk factor in the control group is equal to or less than 55%.

From September 2021 to October 2022 the farm investigations were carried out by the Co-Investigator of the study.

### Feed components

During the herd investigation, information on the feed ingredients used in the rations was collected. Farmers were asked whether they mixed the feed themselves or used a complete feed produced by a feed mill. If complete feed was used the information on the feed label provided by the feed production company for each delivered batch was used to identify the components.

If whey or any other ingredients not listed on the label were added to the diet, this was noted separately during the investigation. In addition, the crude fibre and the dry matter content of the final mix were recorded for the grower and fattening groups, respectively.

### Laboratory analyses feed particle size distribution

Laboratory analyses were conducted on the feed particle size distribution. Samples of the dry feed component were collected from the farms. However, in farms using self-mixing rations, samples could not be taken due to the lower dry matter content of the components. Additionally, some farms had closed systems installed, preventing the collection of dry component samples. The Co-Investigator analyzed the samples at two points: at the midpoint of the sampling period (for all samples collected until then) and at the end of the sampling period (for samples collected from the midpoint to the end) using the same equipment. Two subsamples of equal weight were taken from each sample and individually sieved using a vibrating sieve machine for feed and soil samples (RETSCH GmbH, 42781 Haan, Germany). Sieves with mesh sizes of 4 mm, 2 mm, 1 mm, 0.5 mm, and 0.18 mm were used, resulting in particles found in the collection tray at the bottom measuring < 0.18 mm. To compare the different analyses, results were converted to percentages and grouped into five size categories by diameter (< 4 mm, <2 mm, <1 mm, < 0.5 mm, and < 0.18 mm).

### Laboratory analyses microbiological feed and water quality

Feed and water samples were collected from each herd at the time of the the Co-Investigator`s visit. Feed samples were taken from the mixing vat and the first and last drain pipes if a liquid feed system was established, and from the first and last automatic feeder if a dry feeding system was in place. If whey was part of the diet, a sample was taken from the pipe at the level of the mixing tank. In herds where a death due to HBS had occurred in the previous 7 days, an additional sample was taken from the drain pipe or automatic feeder of the affected pen. Water samples were taken from the first and the last drinkers in the water system. Each drinker was manually cleaned and flamed with a Bunsen burner until an orange discoluoration of the metal was visible, and then the drinker was squeezed with a sterile clamp to allow water to run for five to ten seconds before sampling. The pigs were restrained by the farmer during burning and sampling to prevent contamination of the drinker. Samples were kept out of the sun at room temperature during transport to the laboratory (UFAG LABORATORIEN AG, Sursee, Switzerland) and analysed within one day by microbiological methods. Feed and water samples were screened for the total viable count of aerobes as well as *Enterobacteriaceae* and *E. coli*, respectively, and yeasts and moulds were measured in all feed samples. All results were expressed as colony-forming units per gram (CFU/g). All results were compared with reference values suggested by Kamphues [12].

### Laboratory analyzes fecal samples

Faecal samples were collected from 26 randomly selected herds (ranked randomly by GraphPad with the random number generator) that were examined during this study, 13 herds in the case and the control group, respectively. In each of those 26 herds, ten animals were randomly selected at the start of the study and at least 2 g of native faeces were collected directly from the rectum. The presence of diarrhoea, and vaccination status at the time of the visit, were not considered for the selection of the pigs to be sampled.The vaccination status was part of the questionnaire completed at each farm. Samples were transferred to Eppendorf tubes within 24 h of collection and kept in frozen storage at -80 °C until samples from all farms could be sent together to the Field Station for Epidemiology, University of Veterinary Medicine, Hanover, Germany, for detection of *L. intracellularis*, *B. hyodysenteriae*, and *B. pilosicoli* by quantitative polymerase chain reaction (qPCR). All samples were transported in an insulated box with ice packs to ensure they remained frozen upon arrival at the laboratory.

### Data processing and statistical analysis

The results of the microbiological analysis were transferred to an Excel table (Microsoft Excel, 2010) and logarithmically transformed for further analysis. The distribution of particle sizes in the dry feed samples was converted to percentages, and feed components were categorized as either present (yes) or absent (no) before being added to the same data sheet. All data were then visually checked for completeness and imported into NCSS statistical software (NCSS 2022) for descriptive analysis, visualization, and modeling. Descriptive statistics were performed for each variable after conversion and/or grouping, if necessary. Continuous variables were evaluated for normality both visually and using the Shapiro-Wilk test. Univariable associations with being a case farm were assessed using two-sample t-tests for normally distributed numeric variables, Mann-Whitney U tests for non-normal data, and Chi-square tests for categorical variables. Variables with a p-value of < 0.1 were further described and considered for multivariable analysis.

Analysis of associations was conducted using multivariable logistic regression. The outcome variable was being an HBS case farm (yes or no), treated as a binary variable with exclusive categories. The associations of potential risk factors (with a p-value < 0.1 in univariate analysis) were checked using Pearson’s r for continuous variables and the phi coefficient for categorical variables. None of the potential risk factors were highly correlated (r or phi < 0.7). The model-building strategy involved stepwise backward selection. Initially, a complete model including all potential risk factors was constructed. Nonsignificant variables (*p* < 0.05) were then removed stepwise, checking whether the removal of a variable changed the effect of another variable by more than 20%. This process continued until all variables in the model had either a statistical significance of *p* < 0.05 or importantly changed the effect of another variable. For further analysis of the microbiological results, a multivariable logistic regression test was used. Values from the same sampling point (mixing vat, first drainpipe, last drainpipe, and whey) with a p-value < 0.1 in the univariable analysis were included in the same model, with separate models calculated for each feeding system.

## Data Availability

The present work is part of the study described in Holenweger, F., Schüpbach, G., Hofer, A. et al. Housing and management factors and breed predisposition for haemorrhagic bowel syndrome in swine. Porc Health Manag 9, 44 (2023). 10.1186/s40813-023-00340-y Data supporting the findings of this study are available from the corresponding author on a reasonable request.
